# Effect of moderate beer consumption (with and without ethanol) on osteoporosis in early postmenopausal women: Results of a pilot parallel clinical trial

**DOI:** 10.3389/fnut.2022.1014140

**Published:** 2022-11-15

**Authors:** Marta Trius-Soler, Anna Tresserra-Rimbau, Juan J. Moreno, Pilar Peris, Ramon Estruch, Rosa M. Lamuela-Raventós

**Affiliations:** ^1^Department of Nutrition, Food Sciences and Gastronomy, XIA, School of Pharmacy and Food Sciences, University of Barcelona, Barcelona, Spain; ^2^Nutrition and Food Safety Research Institute (INSA), University of Barcelona, Santa Coloma de Gramanet, Spain; ^3^Centro de Investigación Biomédica en Red (CIBER) de Fisiopatología de la Obesidad y Nutrición, Instituto de Salud Carlos III, Madrid, Spain; ^4^Rheumatology Department, Hospital Clínic, Institut d’Investigacions Biomèdiques August Pi i Sunyer, University of Barcelona, Barcelona, Spain; ^5^Department of Internal Medicine, Hospital Clínic, Institut d’Investigacions Biomèdiques August Pi i Sunyer, University of Barcelona, Barcelona, Spain

**Keywords:** phytoestrogen, polyphenols, alcohol, silicon, bone markers, osteoporosis, menopause

## Abstract

**Introduction:**

Osteoporosis is a chronic progressive bone disease characterized by low bone mineral density (BMD) and micro-architectural deterioration of bone tissue, leading to an increase in bone fragility and the risk of fractures. A well-known risk factor for bone loss is postmenopausal status. Beer may have a protective effect against osteoporosis associated with its content of silicon, polyphenols, iso-α-acids and ethanol, and its moderate consumption may therefore help to reduce bone loss in postmenopausal women.

**Methods:**

Accordingly, a 2-year controlled clinical intervention study was conducted to evaluate if a moderate daily intake of beer with (AB) or without alcohol (NAB) could have beneficial effects on bone tissue. A total of 31 postmenopausal women were assigned to three study groups: 15 were administered AB (330 mL/day) and six, NAB (660 mL/day), whereas, the 10 in the control group refrained from consuming alcohol, NAB, and hop-related products. At baseline and subsequent assessment visits, samples of plasma and urine were taken to analyze biochemical parameters, and data on medical history, diet, and exercise were collected. BMD and the trabecular bone score (TBS) were determined by dual-energy X-ray absorptiometry. Markers of bone formation (bone alkaline phosphatase [BAP] and *N*-propeptide of type I collagen [PINP]) and bone resorption (*N*-telopeptide of type I collagen [NTX] and C-telopeptide of type I collagen [CTX]) were determined annually.

**Results:**

Bone formation markers had increased in the AB and NAB groups compared to the control after the 2-year intervention. However, the evolution of BMD and TBS did not differ among the three groups throughout the study period.

**Discussion:**

Therefore, according to the findings of this pilot study, moderate beer intake does not seem to have a protective effect against bone loss in early post-menopausal women.

## Introduction

Osteoporosis is characterized by low bone mass and micro-architectural deterioration of bone tissue, leading to an increase in bone fragility and risk of bone fractures (1). A major health problem worldwide, this chronic progressive disease constitutes a serious economic burden. The total direct cost of osteoporotic fractures in Europe (excluding the value of quality-adjusted life-years lost) amounted to €56.9 billion in 2019 and 14.8 million women needing osteoporosis treatment were left untreated, generating a treatment gap of 71% (2). The etiology of osteoporosis is multifactorial, and although genetic and hormonal factors strongly influence the rate of bone loss with age, other aspects such as nutrition, lifestyle habits and physical activity also play an important role (1, 3).

Osteoporosis can occur in both sexes but is most frequently observed in postmenopausal women. Estrogen deficiency can increase bone turnover by nearly 90% and the resulting imbalance in bone remodeling leads to a reduction in bone mass and the development of osteoporosis. In women, there are two phases of bone loss: at the onset of menopause, when it can occur at a rapid rate for up to 5 years, and then as a slower aging-related process lasting for 10–20 years, which affects men as well (4). The menopausal transition has also been associated with an accelerated decline in the trabecular bone score (TBS), supporting the thesis that skeletal integrity is particularly at risk at this life stage (5).

Although chronic alcoholism is known to have a negative impact on bone health, beneficial effects on bone tissue have been attributed to a moderate intake of alcohol (3, 6). Thus, bone mineral density (BMD), the gold standard measurement used to diagnose and treat osteoporosis, has been positively associated with alcohol intake in older women in the Framingham Osteoporosis Study (7) and other landmark cohort studies (8). However, only a few studies have compared the effects of different types of alcoholic beverages (e.g., beer, wine, or spirits) on BMD and conflicting results have been obtained (9, 10). In the Framingham Offspring Cohort Study, it was concluded that moderate alcohol intake may be beneficial in postmenopausal women and that beer and wine have a stronger protective effect on BMD compared to spirits, suggesting that beverage constituents other than alcohol may contribute to bone health (11).

The components of beer that may potentiate its protective effects against osteoporosis include silicon, polyphenols, and iso-α-acids. The results of several epidemiological and experimental studies indicate that dietary silicon may increase BMD and reduce bone fragility (12–14). Major sources of silicon in Western diets are cereals/grains and their derivatives, including breakfast cereals, bread, and beer. Other sources are fruits and vegetables (e.g., bananas, raisins, and green beans), as well as unfiltered drinking water. Our exposure to silicon has declined in recent times, due above all to drinking water treatment, cereal processing, and possibly the hydroponic growth of vegetables (15, 16). This would explain why beer is reported to be one of the main sources of dietary silicon in several epidemiological studies, the average content being 6.336 mg/300 mL (14). Moreover, the silicon found in beer is highly bioavailable and most of it is rapidly absorbed and excreted (16–19). Silicon could promote bone formation stimulating cell proliferation and upregulating the expression of osteogenesis gens such as collagen type 1, which is hypothesized to be due to the induction of the extracellular signal-regulated kinases (ERK) pathway. In addition, silicon has been reported to has an influence on both bone remodeling inhibiting the differentiation and activity of osteoclast and early stages of biomineralization (20). Beer is also rich in flavonoids and phytoestrogens (prenylflavonoids) and contains B-vitamins and other minor components (21–23).

Besides the level of bone mass, bone strength is affected by other tissue parameters, such as micro-architecture and the balance and rate of bone remodeling. The TBS evaluates bone texture based on the analysis of lumbar spine dual-energy X-ray absorptiometry (DXA) images and provides information on bone micro-architecture. On the other hand, biochemical markers of bone turnover (BTMs) are products released during bone formation by osteoblasts and bone resorption by osteoclasts, and monitoring their levels is a non-invasive way of assessing bone health. The acceleration of bone turnover after menopause, in which bone resorption outpaces formation, is reflected by an increase in BTMs (approximate 90% increase in resorption markers and 45% in formation markers). This increase correlates with a higher rate of bone loss, especially 5–10 years after menopause and in the trabecular bone. Therefore, BTMs are useful for the prediction of bone loss, assessment of fracture risk, and particularly to monitor the treatment of postmenopausal osteoporosis (1, 24). In clinical practice, the most recommended markers of bone formation are the bone isoform of alkaline phosphatase (BAP) and fragments of type I procollagen released during the formation of type I collagen (*N*-propeptide of type I collagen, PINP). Resorption markers include the fragments released from the telopeptide region of type I collagen following its enzymatic degradation [including the *N*-telopeptide of type I collagen (NTX) and the C-telopeptide of type I collagen (CTX)]. PINP and CTX have been proposed by the International Osteoporosis Foundation as reference markers and the use of at least two BTMs is recommended in clinical studies (24).

To sum up, postmenopausal status is a well-known risk factor related to BMD loss and the development of osteoporosis. Due to the phenolic, silicon and ethanol content of beer, its moderate consumption may help to maintain BMD in postmenopausal women. However, few long-term controlled clinical trials have been performed to evaluate the impact of beer on bone mass (22). To address this lack, we conducted a 2-year controlled clinical intervention study to assess whether a moderate daily intake of alcoholic beer (AB) or non-alcoholic beer (NAB) could have beneficial effects on bone tissue. With this aim, the impact of beer consumption on BTMs was determined and changes in BMD and TBS were monitored in a cohort of postmenopausal women.

## Materials and methods

### Experimental design, study population, and recruitment

This study was a long-term three-arm parallel controlled clinical trial investigating the effect of daily moderate beer consumption on bone tissue. Postmenopausal women aged 45–70 years were recruited into the study from April 2017 to June 2019 from the Outpatient Clinic of the Internal Medicine Department of the Hospital Clinic of Barcelona. The recruitment was done through poster boards in different settings and advertisements on the radio.

The postmenopausal status of each participant was validated by the following criteria: (1) absence of menses in the previous 12 months, during early post-menopausal stage; (2) blood levels of follicle-stimulating hormone (FSH) of 23–116 U/L, and (3) blood levels of 17-β-estradiol (E2) < 37 pg/mL. Women using estrogen therapy or taking silicon or polyphenol supplements were excluded, as were those with known diseases affecting bone metabolism (rheumatoid arthritis, hyperthyroidism, hypercortisolism, renal bone disease, chronic liver disease) or using drugs affecting bone metabolism [fluorides, bisphosphonates, teriparatide or parathormone, strontium ranelate, anabolic steroids, chronic glucocorticoids (>3 months), cytostatics, antiandrogens, and antiepileptics].

Participants were allotted to a study group after a run-in period of 15 days (without consumption of alcoholic drinks, NAB or any hop-related products). The AB group consumed 14 g of ethanol a day in the form of AB (330 mL/day); the NAB group were administered NAB (660 mL/day) containing a similar amount of prenylflavonoid compounds as the AB; and the control group were instructed to refrain from consuming alcohol, NAB or any hop-related products. None of the participants were allowed to consume any alcoholic beverages during the study except what was administered.

Considering the long-term nature of the intervention, participants were assigned to the three study groups according to personal preference, taking into account habitual habits of consumption. As the intervention was dietary, it was blinded to the laboratory personnel and technicians but not to the participants or researchers. During the 2-year intervention, the eligible subjects were asked to visit the research center four times for assessment (at baseline, and 6, 12, and 24 months).

### Ethical statement

The study was conducted in compliance with the Declaration of Helsinki. All procedures were approved by the Bioethics Commission of the University of Barcelona (Institutional Review Board: IRB 00003099) in March 2017, and the study protocol was registered at ISRCTN (ISRCTN13825020). All participants signed informed consent.

### Intervention product characterization and compliance

To standardize the daily consumption of phytoestrogen in each intervention group, the same brand of beer was consumed by all the participants throughout the study. The participants were encouraged to consume beer during meals, which is the recommended dietary practice for alcoholic beverages (25). As NAB has a lower content in polyphenols (26), the NAB intervention was adapted to provide a similar amount of total phytoestrogens as the AB. NAB has also been reported to have lower levels of silicon than lagers, like the one used for the AB intervention. The silicon average content reported by other researchers in NAB (*n* = 6) has been 16.3 (6.4–25.7) mg/L, while in lager AB (*n* = 27) was 23.7 (10.1–56.4) mg/L (27).

Specifically, the women in the study who were administered beer consumed a daily dose of 359 ± 17.4 μg (isoxanthohumol (IX): 302.7 ± 16.8 μg; xanthohumol: 27.9 ± 0.6 μg; 8-prenylnaringenine (8-PN): 5.5 ± 0.4 μg; 6-prenylnaringenine: 22.8 ± 0.3 μg) of prenylflavonoids in the AB (330 mL/day) and 259 ± 10.3 μg [isoxanthohumol (IX): 104.7 ± 3.8 μg; xanthohumol: 81.3 ± 4.0 μg; 8-prenylnaringenine (8-PN): 10.3 ± 0.8 μg; 6-prenylnaringenine: 62.7 ± 2.2 μg] of prenylflavonoids in the NAB (600 mL/day) group. The prenylflavonoid content of the beer was quantified by liquid chromatography coupled to mass spectrometry (LC-MS/MS) in a previous study by Trius-Soler et al. (22, 28), using the methodology of Quifer-Rada et al. with some slight modifications (29).

Intervention compliance was assessed by data obtained from face-to-face interviews, structured dietary questionnaires, and the measurement of IX, a validated biomarker of beer intake. Quantification of IX was carried out in 24-h urine samples collected at baseline, and 6, 12, and 24 months by solid phase extraction LC-MS/MS (30). To facilitate intervention compliance, the participants were supplied with beer every month.

### Measurements and outcome assessment

#### Medical history

Individual information was collected at baseline and updated during each visit by face-to-face interviews. The structured interviews included medical and sociodemographic questions, with special attention given to risk factors for osteoporosis, previous skeletal fractures, menarche and menopause, dietary calcium intake, history of nephrolithiasis, current and past consumption of alcohol and tobacco, and family history of fractures. Sleeping habits, daily life and work stress, time since the onset of menopause, and medication history were also recorded. Participants with serum 25-hydoxyvitamin D (25-OHD) levels < 20 ng/mL were treated with vitamin D supplements, as is usual in clinical practice.

#### Bone mineral density assessment

We assessed the BMD (g/cm^2^) of the lumbar spine, proximal femur (femoral neck and total hip) and whole-body by DXA (GE-LUNAR iDXA Prodigy equipment) at baseline and after 12 and 24 months of intervention. The TBS was calculated using TBS iNsight software (V1.8) (Medimaps Group, Geneva, Switzerland) on the DXA lumbar spine images. Osteoporosis was defined by T-score values ≤−2.5 at the lumbar spine and/or proximal femur according to the WHO criteria and a TBS value < 1.230 indicated degraded micro-architecture (31, 32). BMD assessment was performed following standardized scanning protocols by the CETIR medical group (CETIR Grup Mèdic, Barcelona, Spain).

#### Anthropometric measurements and body composition

Anthropometric measurements (height, weight, and waist circumference) were obtained at each visit by trained registered staff following anthropometric standardization protocols. Weight was determined using a high-quality calibrated scale, with the participants wearing light clothing and no shoes. Height was measured with a wall-mounted stadiometer. Body mass index (kg/m^2^) (BMI) was calculated as weight (kg) divided by height squared (m^2^). Waist circumference was measured using an inelastic flexible tape positioned at the midpoint between the lower margin of the last palpable rib and the top of the iliac crest (33).

Total body and regional body composition were estimated using DXA. Lean mass (kg) and fat mass (kg) were both indexed to height to create the fat mass index (kg/m^2^) and lean mass index (kg/m^2^). Measurements were assessed by the CETIR medical group (CETIR Grup Mèdic, Barcelona, Spain).

#### Biological samples and biochemical analyses

Overnight fasting blood samples and morning spot urine (between 8–9 a.m., to control circadian cycles) were collected at baseline and 6, 12, and 24 months of intervention. Automated biochemical profiles were measured at the Biomedical Diagnostic Center of the Hospital Clinic. The lower detection limits of plasma E2 was 12 pg/mL. Levels below these limits were defined as 11 pg/mL. 24-h urine samples were also collected at all visits and stored in aliquots at −80°C until analyzed for IX, the biomarker of intervention compliance.

Serum BAP was measured by ELISA (immunodiagnostic Systems, Boldom, UK), and serum CTX and PINP by a Cobas e601 analyzer (Roche Diagnostics, Mannheim, Germany). Urinary NTX was measured by ELISA (Osteomark NTX-I, Alere, Scarborough, ME, USA) and expressed as a ratio to creatinine. Plasma parathyroid hormone (PTH) and serum 25-OHD were determined by Atellica Solution (Siemens Healthineers, Tarrytown, NY, USA) and a Liaison analyzer (DiaSorin, Saluggia, Italy), respectively. A concentration of 25-OHD < 20 ng/mL was considered to be vitamin D deficiency. Blood and urine samples were obtained between 8:00 and 9:00 a.m. after overnight fasting.

#### Dietary intake and physical activity assessments

Dietary intake over the previous 12 months was assessed by trained staff at baseline, the halfway point (12 months) and at the end (24 months) of the study using a validated 151-item semi-quantitative food frequency questionnaire (FFQ) (34). Total energy intake (kcal/day) and absolute consumption values of coffee (with caffeine) and tea per day were estimated according to Spanish food composition tables (34). Calcium and vitamin D intake were also estimated by the 151-item FFQ. Total polyphenol intake (mg/day) was estimated by multiplying the polyphenol content in each food item (data obtained from the Phenol-Explorer database) by the daily consumption of the food item according to the FFQ (35). In addition, the 14-point Mediterranean Diet Adherence questionnaire was used as an overall diet quality index to evaluate differences between study groups at baseline (36).

Physical activity was monitored at the four intervention visits. It was measured as the metabolic equivalent of task per day (MET-min/day) using the Minnesota leisure-time questionnaire, previously validated in a population of Spanish women (37).

### Sample size calculation

In postmenopausal women, rates of spine and hip bone loss are 0.022 g/cm^2^ per year (2.0%) and 0.013 g/cm^2^ per year (1.4%), respectively (38). For a parallel design and an analysis of repeated measures, statistical power calculation indicated that to recognize as statistically significant a difference greater than or equal 0.020 g/cm^2^ (2.0%) in total hip BMD with a common standard deviation of 0.025 g/cm^2^, assuming a maximum loss of 10% of participants, and a correlation coefficient between the initial and final measurements as 0.7; 17 subjects per group will be needed to complete the study (α = 0.05; power = 0.8).

### Statistical analyses

Continuous variables were expressed as median (Q1–Q3). Categorical variables were expressed as number (*n*) and proportion (%). Differences in the characteristics of volunteers between groups at baseline were tested by the chi-square test for categorical variables and the Kruskal–Wallis test followed by the *post-hoc* Dunn’s test for continuous variables.

The effect of the interventions on bone turnover and bone health markers was estimated by performing a generalized estimating equation on gamma regression models for repeated measures (identity link function, autoregressive of order correlation, and robust standard error parameters were specified). Adjusted differences and their corresponding 95% confidence intervals were computed using increasing complexity models. A time-exposure interaction term allowed the evaluation of potential differences between intervention groups in response to changes over time. Spearman’s correlations were used to summarize the relationship between the BTMs and the BMD values at baseline and annually.

The % relative changes for bone turnover and bone health markers were calculated. Intergroup differences between baseline, and at 12 and 24 months were analyzed by a non-parametric test for two related samples in each study arm. A Wilcoxon matched-pair signed-rank test for small samples was applied to symmetric variables, and the sign test of matched pairs was used for asymmetric variables. Symmetry was studied by the skewness and kurtosis test for normality (control and AB group) or graphically (NAB group).

Intergroup differences in relevant clinical and anthropometric measurements as well as in dietary patterns between baseline and 24 months were also analyzed by a non-parametric test for two related samples in each study arm. Intragroup differences in dietary patterns were assessed by a Kruskal–Wallis test followed by *post-hoc* Dunn’s test in each group.

All statistical analyses were conducted using the Stata statistical software package version 16.0 (StataCorp, College Station, TX, USA). Statistical tests were two-sided and *p-*values below 0.05 were considered significant. Figures were performed using the Prism 9.0.0 software package.

## Results

### Study subjects, intervention, and compliance

**FIGURE 1:**
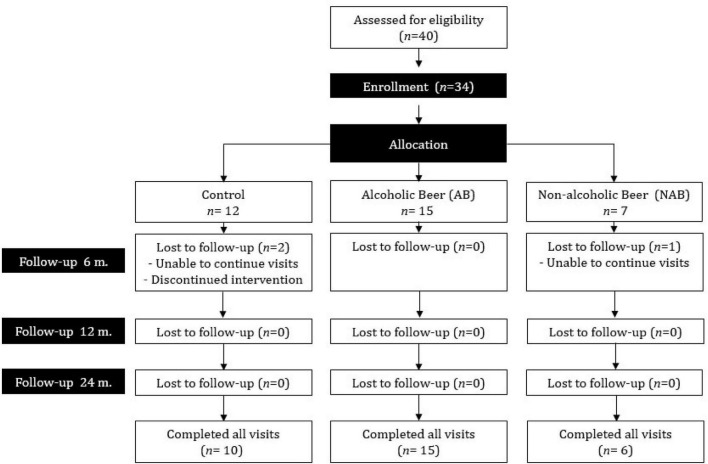
Flow diagram of participant recruitment and compliance in each phase of the intervention trial.

Of the 34 postmenopausal women enrolled at baseline, 31 completed the outcome assessments at 12 and 24 months (Figure 1). Of the women that finished the intervention, 15 had chosen to be in the AB group, six in the NAB group, and 10 in the control group. The drop-outs were due to difficulties with continuing the assessment visits or complying with the assigned intervention, as reported by the participant. Otherwise, subject compliance with the intervention was 100% according to dietary self-records and interviews. To confirm intervention adherence, IX concentrations were measured in the 24-h urine provided by the participants at baseline, and 6, 12, and 24 months, thus participants could drink beer at any time of the day but were encouraged to do it with meals. At baseline, IX concentration was below the detection limit (<0.04 ppb) for 71.0% of the urine samples. At follow-up visits (6, 12, and 24 months), IX values confirmed intervention compliance in 96.7, 97.8, and 77.8% of urine samples of the control, AB, and NAB groups, respectively. The concentration of IX was highly variable among samples.

### Participant characteristics at baseline

Tables 1, 2 summarize the clinical, anthropometric, densitometric and biochemical parameters of the trial participants. Briefly, the volunteers had a median (Q1, Q3) age of 55 (53–58) years and a BMI of 26.3 (24.7–29.0) kg/m^2^. Most were normo-weight or overweight with an elevated waist circumference (Table 2). Although median baseline values of BTMs were within the reference ranges in all three groups, Q1–Q3 values were in the upper reference range or higher (Table 2; 39). Two participants (one in the control and the other in the AB group) presented densitometric osteoporosis in the lumbar spine at baseline.

**TABLE 1:** Baseline characteristics, bone turnover markers and dual-energy X-ray absorptiometry (DXA) parameters of the participants according to the intervention group.

	Control (*n* = 10)	AB (*n* = 15)	NAB (*n* = 6)	*p-*value
**Medical history records**				
Age, years	55 (53–59)	54 (53–56)	57 (54–59)	0.614
Time since the onset of menopause, months	50.0 (18.0–96.0)	24.0 (15.0–48.0)	22.5 (15.0–50.0)	0.553
Previous fractures (after 45 years), n (%)	2 (20.0)	2 (13.3)	1 (16.7)	0.605
Family history of fractures, *n* (%)	1 (10.1)	1 (6.7)	5 (83.3)	** < 0.001**
Early menopause, *n* (%)	0 (0.0)	1 (6.3)	1 (16.7)	0.421
**Lifestyle habits**				
Smoking habit, *n* (%)				
Current	0 (0.0)	6 (40.0)	2 (33.3)	0.112
Former	3 (30.0)	4 (26.7)	0 (0.0)	
Never	7 (70.0)	5 (33.3)	4 (66.7)	
Sleeping time, hours	6.0 (6.0–7.0)	7.0 (6.5–8.0)	7.3 (6.0–7.5)	0.193
^1^Stress/depression from daily life	2.5 (1.0–3.0)^ab^	3.0 (3.0–4.0)^a^	1.5 (1.0–2.0)^b^	**0.025**
^1^Stress/depression from work	2.5 (2.0–3.0)	3.0 (2.0–4.0)	2.5 (1.0–4.0)	0.905
Physical activity, MET-min/day	840 (480–1,146)	552 (304–807)	460 (396–601)	0.238
**DXA parameters**				
Lumbar spine				
BMD, g/cm^2^	1.05 (0.95–1.17)	1.01 (0.99–1.14)	1.07 (1.00–1.16)	0.861
T-score	−0.95 (−1.90, −0.29)	−1.56 (−1.76, −0.50)	−1.06 (−1.40, −0.35)	0.876
TBS	1.41 (1.35–1.47)^a^	1.33 (1.28–1.35)^b^	1.33 (1.30–1.41)^ab^	0.021
**Femoral neck**				
BMD, g/cm^2^	0.91 (0.86–1.03)	0.86 (0.75–0.90)	0.80 (0.77–0.92)	0.218
T-score	−0.61 (−1.00, 0.42)	−0.98 (−1.93, 0.65)	−1.36 (−1.77, −0.50)	0.226
**Total hip**				
BMD, g/cm^2^	0.99 (0.94–1.07)	0.89 (0.85–1.01)	0.88 (0.81–1.01)	0.099
T-score	−0.06 (−0.48, 0.610)	−0.92 (−1.27, 0.08)	−1.18 (−1.59, 0.06)	0.094
**Whole body**				
BMD, g/cm^2^	1.07 (1.00–1.11)	1.04 (0.98, 1.09)	1.07 (0.99, 1.10)	0.737
T-score	0.20 (−0.50, 0.50)	−0.20 (−0.80, 0.30)	0.20 (−0.10, 0.40)	0.522
**Bone turnover markers**				
^2^BAP, ng/mL	12.6 (10.1–14.3)	12.2 (10.5–14.8)	11.8 (9.6–15.4)	0.932
^3^PINP, ng/mL	55.5 (43.2–66.4)	55.5 (44.8–64.0)	43.8 (34.7–81.4)	0.724
^4^NTX, nMol/nMol	60.5 (53.0–74.0)	66.0 (45.0–74.0)	49.5 (47.0–55.0)	0.360
^5^CTX, ng/mL	0.54 (0.51–0.75)	0.52 (0.44–0.68)	0.43 (0.31–0.67)	0.320
**Medication, *n* (%)**				
Antihypertensive agents	0 (0.0)	3 (20.0)	1 (16.7)	0.328
Lipid-lowering medication	0 (0.0)	2 (13.3)	0 (0.0)	0.320
Antidepressants, sedatives, anxiety pills	2 (20.0)	3 (20.0)	1 (16.7)	0.983
Dietary supplements	4 (40.0)	8 (53.3)	2 (33.3)	0.653

^1^Score from 1–5.

^2^BAP reference values: 6.0–13.8 ng/mL.

^3^PINP reference values: 20.8–60.6 ng/mL.

^4^NTX reference values: 19.3–68.9 nMol/nMol.

^5^CTX reference values: 0.14–0.48 ng/mL. AB, alcoholic beer; BAP, bone alkaline phosphatase; BMD, bone mineral density; CTX, C-telopeptide of type I collagen; NAB, non-alcoholic beer; NTX, *N*-telopeptide of type I collagen; PINP, *N*-propeptide of type I collagen; TBS, trabecular bone score.

Categorical variables are expressed as number (*n*) and proportion (%).

Chi-square test was applied to study differences in categorical variables.

Continuous variables are presented as median values (Q1–Q3).

Kruskal–Wallis test followed by *post-hoc* Dunn’s test were applied to study differences in continuous variables. Medians within the same row carrying different superscripts (a, b) are significantly different.

*p*-value < 0.05. The bold values represent the *p*-value < 0.050 is considered statistically significant.

**TABLE 2:** Baseline anthropometric measurements, dietary history, and biochemical analyses of the participants according to intervention group.

	Control (*n* = 10)	AB (*n* = 15)	NAB (*n* = 6)	*p*-value
**Anthropometric measures**				
BMI, kg/m^2^	26.5 (25.3–32.5)	26.5 (23.1–28.6)	25.3 (24.7–29.0)	0.595
WC, cm	90.0 (85.5–100.0)	88.7 (79.5, 96.4)	84.5 (80.3–90.1)	0.588
Body fat mass, %	44.1 (40.2–45.1)	42.7 (39.2–47.5)	40.3 (39.1–48.2)	0.900
Fat mass index, kg/m^2^	11.5 (9.6–15.3)	11.2 (8.8–13.0)	10.5 (9.3–12.3)	0.636
Lean mass index, kg/m^2^	15.0 (14.5–17.2)^a^	14.2 (13.1–14.6)^b^	14.6 (14.0–16.5)^ab^	0.034
**Dietary history**				
Total energy intake, kcal/day	2,699 (2,556–3,022)	2,599 (2,127–3,138)	2,348 (2,268–2,682)	0.320
Protein intake, % kcal/daily kcal	20.4 (16.3–20.9)	19.2 (17.4–21.8)	18.1 (16.9–20.4)	0.781
Calcium intake, mg/day	1,365 (1,090–15,679)	1,199 (935–1,552)	1,083 (824–1,334)	0.405
Vitamin D intake, μg/day	6.1 (4.0–9.8)	6.4 (4.9–8.3)	6.3 (5.7–7.0)	0.968
Total polyphenol intake, mg/day	1,064 (770–1,419)	753 (487–853)	830 (677–1,450)	0.127
**Alcohol drinking habit**				
Weekly, *n* (%)	1 (10.0)	9 (60.0)	1 (16.7)	0.061
Occasionally, *n* (%)	7 (70.0)	6 (40.0)	4 (66.7)	
Never, *n* (%)	2 (20.0)	0 (0.00)	1 (16.7)	
**Type of alcohol preferred**				
Beer, *n* (%)	3 (30.0)	7 (46.7)	3 (50.0)	0.419
Wine, *n* (%)	4 (40.0)	7 (46.7)	2 (33.3)	
Spirits, *n* (%)	0 (0.0)	1 (6.7)	0 (0.0)	
None, *n* (%)	3 (30.0)	0 (0.0)	1 (16.7)	
MedDiet, 14-item score	9.0 (7.0–9.5)	7.0 (6.0–9.0)	8.5 (7.0–10.0)	0.338
Tea consumption, g/day	14.3 (0.0–21.4)	7.1 (0.0–21.4)	1.7 (0.0– 50)	0.839
Caffeinated coffee consumption, g/day	50 (21–125)	50 (0–125)	88 (0–125)	0.757
**Biochemical markers**				
Creatinine, mg/dL	0.71 (0.56–0.83)	0.64 (0.59–0.75)	0.68 (0.66–0.69)	0.456
Calcium (serum), ng/dL	9.3 (9.0–9.5)	9.3 (9.0–9.5)	9.3 (9.1–9.5)	0.969
PTH, ng/mL	63.0 (44.0– 80.0)	52.0 (46.0–69.0)	66.5 (46.0–73.0)	0.751
25-hydroxyvitamin D, ng/mL	23.7 (20.6–26.5)	25.4 (18.6–35.7)	24.6 (14.1–38.6)	0.743
FSH, U/L	75.0 (56.3–84.3)^ab^	88.5 (74.0–105.5)^b^	70.4 (37.2–72.2)^a^	0.027
E2, pg/mL	23.0 (15.0–31.0)	18.0 (13.5–25.0)	22.0 (21.0–25.0)	0.587
TSH, ng/mL	2.15 (0.94–3.75)	1.82 (1.48–2.96)	2.56 (2.19–2.91)	0.695
FT4, ng/mL	1.17 (1.11–1.32)	1.13 (1.02–1.26)	1.08 (0.98–1.10)	0.159
T3, ng/mL	1.23 (0.98–1.41)	1.14 (1.06–1.25)	1.09 (0.93–1.19)	0.634
AST, U/L	19 (18–20)^a^	23 (19–25)^b^	20 (17–25)^ab^	**0.028**
ALT, U/L	16 (14–18)	18 (16–28)	18 (13–22)	0.191
GGT, U/L	13 (10–14)^a^	22 (14–26)^b^	14 (12–23)^ab^	**0.044**

AB, alcoholic beer; ALT, alanine transaminase; AST, aspartate transaminase; BMI, body mass index; E2, 17-β-estradiol; FSH, follicle-stimulating hormone; FT4, thyroxine; GGT, gamma-glutamyl transferase; MedDiet, mediterranean diet adherence screener 14-item score; NAB, non-alcoholic beer; PTH, parathyroid hormone; T3, triiodothyronine; TSH, thyroid stimulating hormone; WC, waist circumference.

Categorical variables are expressed as number (*n*) and proportion (%).

Chi-square test was applied to study differences in categorical variables.

Continuous variables are presented as median values (Q1–Q3).

Kruskal–Wallis test followed by *post-hoc* Dunn’s test were applied to study differences in continuous variables.

Medians within the same row carrying different superscripts (a, b) are significantly different.

*p*-value < 0.05. The bold values represent the *p*-value < 0.050 is considered statistically significant.

In terms of absolute analytical values, serum creatinine and calcium concentrations were within the reference ranges, while PTH serum levels were within the reference range or above. Median levels of 25-OHD for each group were above 20 ng/mL (with nine subjects showing values < 20 ng/mL: 2 control, 4 AB, and 3 NAB, respectively) (Table 2). Taking as a reference the results reported for women aged 60 years or more in a cohort study of 5,629 healthy Caucasian men and women (15–98 years), the participants in the present study had similar or higher indices of mean body fat (%) and body fat mass, and a lower lean mass index (40).

Significant differences in baseline characteristics between treatment arms were only observed in family history of fractures, daily life-induced stress/depression score, TBS values (higher in the control group), lean mass index values, FSH levels, aspartate transaminase (AST) and gamma-glutamyl transferase (GGT) (Tables 1, 2). No significant differences were observed in baseline DMD values in any skeletal location or in baseline BTMs between groups. Five out of 6 (83.3%) of the volunteers in the NAB group had a family history of fractures, whereas women in the AB group reported higher levels of stress in their daily life (Table 1). At baseline, median FSH levels of the AB group were significantly higher compared to the NAB group, while the lean mass index was lower in the AB than the control group (Table 2). Moreover, the AB group normally drank alcoholic beverages more often (60% reported a weekly frequency habit) and had significantly higher levels of AST and GGT compared to the control group, but within the reference range (Table 2).

Four women (13%) were taking antihypertensive medication, 2 (6%) antihyperlipidemic medication, 6 (20%) antidepressants/sedative/anxiety pills, and 14 (45%) dietary supplements. No statistical differences in medication use were observed between groups at baseline or at the end of the intervention (Table 1).

### Controlled covariates: Anthropometric, clinical, and dietary intake changes during follow-up

For a more in-depth study of the intervention effects on bone tissue, changes in anthropometric and biochemical variables that might explain or modify these effects were monitored (Supplementary Table 1). At the end of the intervention (24 months), both fat and lean mass indices had significantly increased in the AB group; accordingly, the BMI was also higher, although not significantly. Additionally, median (Q1–Q3) creatinine levels had significantly increased in the control and AB groups, whereas PTH levels increased significantly only in the AB group.

Changes in individual dietary patterns during follow-up were also monitored (Supplementary Table 2). Regarding the median dietary pattern of the participants, intake was low for carbohydrates (<45–60% kcal/total kcal) and high for sugar (>10% kcal/total kcal), protein (>12–15% kcal/total kcal), fat (>20–35% kcal/total kcal), and saturated fatty acids (<10% kcal/total kcal), according to the reference values of the European Food Safety Authority (41). Fiber intake met the EFSA recommendations and alcohol consumption ranged from low to moderate. Calcium intake also met the recommendation for older people (750 mg/day) or was slightly below, whereas the intake of dietary vitamin D was below the level established for adults (600 IU/day or 15 μg/day) (41).

According to the FFQ data, alcohol consumption at baseline and throughout the study period (due to the intervention) was significantly higher in the AB than in the NAB and control groups. Median (Q1–Q3) percentages of energy provided by carbohydrate and fat intake were significantly higher in the NAB group during follow-up. The percentage of energy provided by simple sugar in the NAB group was also higher than in the AB group at 12 and 24 months. Dietary factors within the NAB group did not change significantly during the study, whereas, at 24 months a significant reduction in the percentage of energy intake from carbohydrates was reported by the AB group and from saturated fatty acids by the control group, the latter also reporting a significantly lower intake of calcium.

### Changes in bone turnover markers according to beer consumption

**FIGURE 2:**
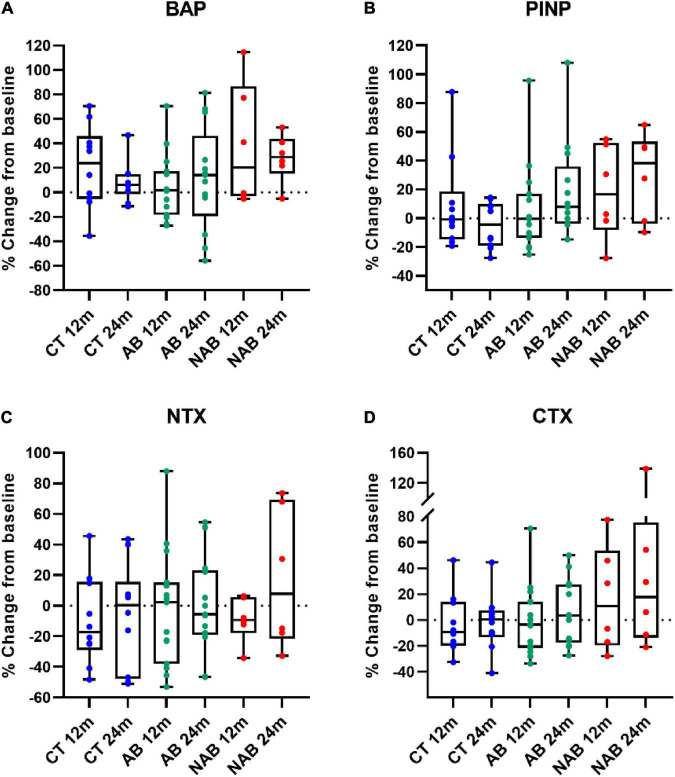
Relative change at 12 months (12 m) and 24 months (24 m) in **(A)** bone alkaline phosphatase (BAP); **(B)** *N*-propeptide of type I collagen (PINP); **(C)** *N*-telopeptide of type I collagen (NTX); **(D)** C-telopeptide of type I collagen (CTX) of the control (CT), alcoholic beer (AB) and non-alcoholic beer (NAB) group in comparison to baseline. Median (min, max) values are illustrated. No significant differences were found between baseline and 12 or 24 months in each arm. Matched-pair signed-rank test was used for statistical intragroup comparisons throughout the intervention. Sing-test of matched pairs was used in asymmetric distributed variables.

Prespecified endpoints were changes in bone formation and bone resorption markers at 12 and 24 months compared to baseline in each group (Figure 2). PINP values in the AB and NAB groups had increased at 24 months but did not change in the control group. All groups displayed a high inter-variability in % changes from baseline.

Table 3 shows the intervention effect on BTMs at follow-up. At 24 months, postmenopausal women consuming AB and NAB exhibited a significantly higher increase in PINP than those in the control group. The linearly measured time-exposure interaction was found to be statistically significant when comparing PINP values of the AB and control groups (*p-*trend: 0.029) and the NAB and control groups (*p-*trend: 0.001); the adjusted differences in PINP levels were expected to increase by 0.39 ng/mL (95% CI: 0.04, 0.74) and 0.76 ng/mL (95% CI: 0.31, 1.21) for every 12 additional months of intervention, respectively. Additionally, the mean difference in BAP values between baseline and 24 months was significantly higher in the NAB than in the control group, with a significant linear time–exposure interaction (adjusted difference: 0.09; 95% CI: 0.01, 0.17; *p-*trend: 0.026). In contrast, no significant changes in the NTX and CTX bone resorption markers were observed in either of the intervention groups compared to the control. The alcoholic fraction derived from AB consumption appeared to have an opposite effect on BAP levels compared to the non-alcoholic fraction of beer at 24 months of intervention (AB vs. NAB group, Table 3).

**TABLE 3:** Intervention effect on bone formation and bone resorption markers at follow-up.

	AB vs. control			NAB vs. control			AB vs. NAB		
	Difference time-exposure (95% CI)	*p*-value	*p*-trend	Difference time-exposure (95% CI)	*p*-value	*p*-trend	Difference time-exposure (95% CI)	*p*-value	*p*-trend
**BAP, ng/mL**									
Model 1	−0.4 (−3.2, 2.3)	0.748	0.722	2.1 (−0.0, 4.2)	0.053	0.065	−2.5 (−5.7, 0.6)	0.109	0.088
Model 2	−0.4 (−2.8, 1.9)	0.71	0.653	1.9 (0.2, 3.5)	**0.033**	**0.023**	−2.3 (−4.9, 0.3)	0.08	**0.041**
Model 3	−0.9 (−3.1, 1.4)	0.452	0.423	1.8 (0.1, 3.5)	**0.039**	**0.026**	−2.6 (−5.1,−0.1)	**0.038**	**0.019**
**PINP, ng/mL**									
Model 1	9.9 (1.0, 18.8)	**0.03**	**0.036**	16.7 (3.9, 29.6)	**0.011**	**0.016**	−6.8 (−19.1, 5.5)	0.279	0.315
Model 2	10.6 (2.1, 19.0)	**0.014**	**0.02**	18.2 (7.7, 28.7)	**0.001**	**0.001**	−7.6 (−17.6, 2.4)	0.135	0.146
Model 3	9.5 (1.5, 17.5)	**0.019**	**0.029**	17.9 (7.7, 28.1)	**0.001**	0.001	−8.4 (−18.7, 1.9)	0.111	0.12
**NTX, nMol/nMol**									
Model 1	4.5 (−14.4, 23.3)	0.641	0.68	12.0 (−10.5, 34.6)	0.327	0.327	−7.6 (−26.6, 11.5)	0.793	0.456
Model 2	5.7 (−11.5, 22.9)	0.516	0.561	9.6 (−11.8, 30.9)	0.381	0.416	−3.9 (−21.7, 14.0)	0.672	0.693
Model 3	3.0 (−13.3, 19.2)	0.721	0.743	8.6 (−12.0, 29.3)	0.413	0.443	−5.7 (−23.5, 12.2)	0.533	0.561
**CTX, ng/mL**									
Model 1	−0.01 (−0.11, 0.08)	0.92	0.891	0.80 (−0.08, 0.24)	0.327	0.343	−0.08 (−0.24, 0.07)	0.429	0.289
Model 2	0.00 (−0.10, 0.10)	0.977	0.972	0.11 (−0.02, 0.25)	0.111	0.098	−0.11 (−0.24, 0.02)	0.098	0.096
Model 3	0.01 (−0.10, 0.10)	0.983	0.958	0.11 (−0.02, 0.25)	0.104	0.11	−0.11 (−0.25, 0.02)	0.095	0.093

AB, alcoholic beer; BAP, bone alkaline phosphatase; CI, coefficient interval; CTX, C-telopeptide of type I collagen; NAB, non-alcoholic beer; NTX, *N*-telopeptide of type I collagen; PINP, *N*-propeptide of type I collagen.

Generalized estimating equation (GEE) models to estimate the effect (difference group × time 95% CI) on the intervention between the intervention groups and the control group.

Model 1: adjusted by age at baseline; Model 2: adjusted like Model 1 plus time since the onset of menopause, follicle-stimulating hormone concentration, smoking habit, lean mass index at baseline; Model 3: adjusted like Model 2 plus total energy intake, physical activity as MET-min/day, and calcium dietary intake at baseline.

*p*-value: group × time interaction; *p*-trend: group × time interaction (continuous).

Two participants of the AB group were excluded from the analysis at 24 months due to traumatic fractures during the last year of the intervention.

*p*-value < 0.05. The bold values represent the *p*-value < 0.050 is considered statistically significant.

Among all participants, % changes in PINP were positively correlated with % changes in BAP at 12 (*r*: 0.568; *p-*value: 0.001) and 24 months (*r*: 0.560; *p-*value: 0.002) from baseline. Moreover, % changes in CTX were also correlated with% changes in PINP levels at 12 (*r*: 0.689; *p*-value: < 0.001) and 24 months (*r*: 0.556; *p*-value: 0.002), and BAP levels at 24 months (*r*: 0.381; *p*-value: 0.042). Furthermore, % changes in resorption markers were positively correlated at 24 months from baseline (*r*: 0.375; *p-*value: 0.045).

### Changes in bone mass and trabecular bone score according to beer consumption

**FIGURE 3:**
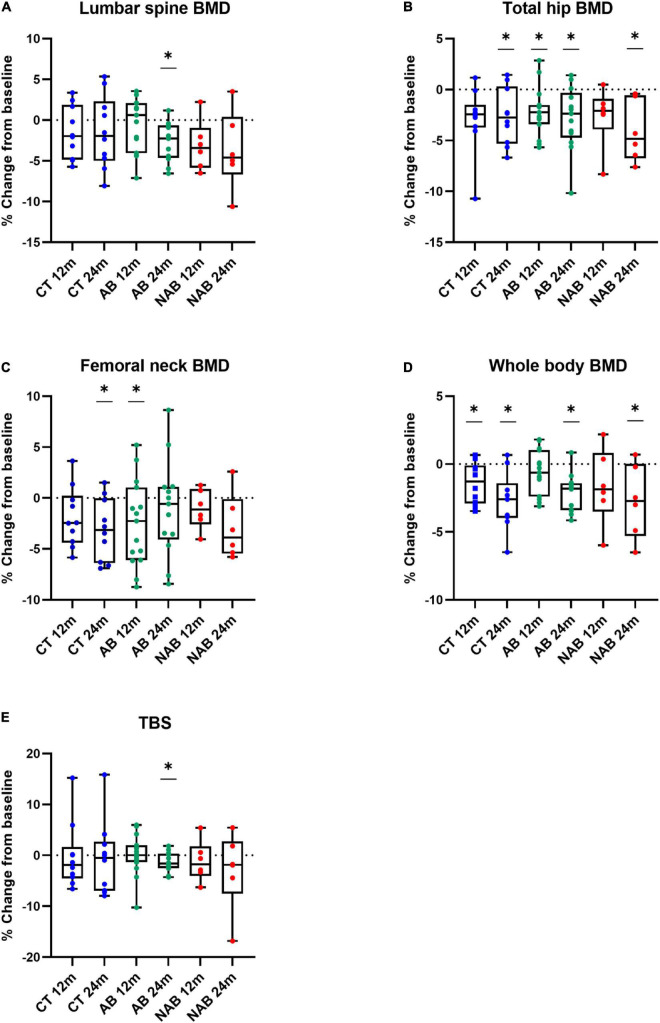
Relative change at 12 months (12 m) and 24 months (24 m) in **(A)** Lumbar spine bone mineral density (BMD). **(B)** Total hip BMD. **(C)** Femoral neck BMD. **(D)** Whole body BMD. **(E)** Trabecular bone score (TBS) of the control (CT), alcoholic beer (AB) and non-alcoholic beer (NAB) group in comparison to baseline. Median (min, max) values are illustrated. (*) refers to the difference between baseline and 12 or 24 months in each arm. Matched-pair signed-rank test was used for statistical intragroup comparisons throughout the intervention. Sing-test of matched pairs was used in asymmetric distributed variables.

Prespecified endpoints also included changes in BMD and TBS. Figure 3 shows % changes in lumbar spine, total hip, femoral neck, and whole-body BMD as well as % changes in TBS at 12 and 24 months from baseline values in the three study groups. As shown in the figure, total hip and whole-body BMD significantly decreased in all groups during the 2-year study period. Additionally, a significant decrease in the femoral neck BMD was observed in the control group and in lumbar spine BMD and TBS in the AB group at 24 months.

The intervention effect on BMD and TBS was analyzed considering exposure time interactions (Table 4). Changes in bone health according to DXA measurements were not significantly different when comparing the AB or NAB group with the control; no significant differences were found between the beer interventions either.

**TABLE 4:** Intervention effect on bone mineral density (BMD) and bone quality at follow-up.

	AB vs. control			NAB vs. control			AB vs. NAB		
	Difference time-exposure (95% CI)	*p*-value	*p*-trend	Difference time-exposure (95% CI)	*p*-value	*p*-trend	Difference time-exposure (95% CI)	*p*-value	*p*-trend
**Lumbar spine BMD, g/cm^2^**									
Model 1	−0.01 (−0.04, 0.02)	0.556	0.599	−0.02 (0.07, 0.02)	0.313	0.324	0.01 (−0.02, 0.05)	0.477	0.464
Model 2	−0.01 (−0.04, 0.02)	0.546	0.586	−0.03 (−0.07, 0.02)	0.258	0.267	0.02 (−0.02, 0.05)	0.393	0.384
Model 3	−0.01 (−0.04, 0.02)	0.484	0.535	−0.03 (−0.07, 0.02)	0.237	0.247	0.02 (−0.02, 0.05)	0.405	0.389
**Femoral neck BMD, g/cm^2^**									
Model 1	0.01 (−0.01, 0.04)	0.337	0.408	0.00 (−0.03, 0.03)	0.87	0.881	0.01 (−0.02, 0.04)	0.47	0.539
Model 2	0.01 (−0.01, 0.04)	0.329	0.386	0.01 (−0.02, 0.03)	0.687	0.687	0.01 (−0.02, 0.04)	0.624	0.693
Model 3	0.01 (−0.02, 0.04)	0.392	0.478	0.01 (−0.02, 0.03)	0.711	0.715	0.01 (−0.02, 0.04)	0.675	0.763
**Total hip BMD, g/cm^2^**									
Model 1	−0.00 (−0.02, 0.02)	0.976	0.904	−0.01 (−0.04, 0.01)	0.378	0.371	0.01 (−0.01, 0.04)	0.387	0.42
Model 2	0.00 (−0.02, 0.02)	0.961	0.893	−0.01 (−0.04, 0.02)	0.49	0.484	0.01 (−0.02, 0.04)	0.501	0.536
Model 3	−0.00 (−0.02, 0.02)	0.945	0.864	−0.01 (−0.04, 0.02)	0.519	0.511	0.01 (−0.02, 0.03)	0.532	0.578
**Whole body BMD, g/cm^2^**									
Model 1	0.00 (−0.01, 0.02)	0.346	0.322	0.00 (−0.02, 0.03)	0.916	0.913	0.01 (−0.02, 0.03)	0.564	0.538
Model 2	0.01 (−0.01, 0.02)	0.406	0.374	−0.00 (−0.03, 0.02)	0.952	0.956	0.01 (−0.01, 0.03)	0.519	0.492
Model 3	0.01 (−0.01, 0.02)	0.446	0.408	−0.00 (−0.03, 0.02)	0.974	0.977	0.01 (−0.02, 0.03)	0.558	0.525
**TBS**									
Model 1	−0.00 (−0.06, 0.05)	0.952	0.978	−0.03 (−0.12, 0.07)	0.574	0.572	0.03 (−0.05, 0.10)	0.524	0.507
Model 2	−0.00 (−0.06, 0.05)	0.882	0.909	−0.03 (−0.12, 0.07)	0.541	0.54	0.03 (−0.05, 0.10)	0.527	0.509
Model 3	−0.01 (−0.06, 0.05)	0.821	0.847	−0.03 (−0.12, 0.06)	0.546	0.544	0.02 (−0.05, 0.10)	0.574	0.555

AB, alcoholic beer; CI, coefficient interval; NAB, non-alcoholic beer; TBS, trabecular bone score.

Generalized estimating equation (GEE) models to estimate the effect (difference group × time 95% CI) on the intervention between the intervention groups and the control group.

Model 1: adjusted by age at baseline; Model 2: adjusted like Model 1 plus time since the onset of menopause, follicle-stimulating hormone concentration, smoking habit, lean mass index at baseline; Model 3: adjusted like Model 2 plus total energy intake and physical activity as MET-min/day at baseline.

*p*-value: group × time interaction; *p*-trend: group × time interaction (continuous).

Two participants of the AB group were excluded from the analysis at 24 months due to traumatic fractures during the last year of the intervention.

*p*-value < 0.05.

When we analyzed the % of subjects with a decrease in BMD > 3% in either lumbar spine, total hip, and femoral neck at 12 and 24 months, no significant differences were observed between the three groups.

## Discussion

In this 2-year parallel controlled clinical trial with postmenopausal women, AB and NAB consumption was found to increase bone formation markers (i.e., PINP in both intervention groups and BAP only in the NAB group) in comparison with the control group. Nevertheless, DXA scans revealed that neither AB nor NAB interventions attenuated expected postmenopausal BMD and TBS loss, a finding that could be partly attributed to the relatively early postmenopausal stage of the participants (mean age of 55 years), when the menopause-related increase in bone turnover tends to be higher.

The effects of beer or specific beer components on BMD loss have been previously reported (22). Excessive alcohol consumption is associated with a higher risk of osteoporotic fractures (42) and an imbalance in bone remodeling, which becomes skewed toward bone loss (43). Beyond this well-documented association, the effects of moderate alcohol drinking on bone health have also been studied. A recent meta-analysis by Godos et al. (42) found that up to two standard alcoholic drinks/day vs. alcohol abstinence are related with a higher lumbar and femoral neck BMD, while up to one standard drink/day was found to be associated with higher hip BMD (42). In the Framingham Offspring Cohort Study, the relationship between alcohol intake and BMD at three hip sites and the lumbar spine was analyzed in 1,289 postmenopausal and 298 premenopausal women (11). The main conclusion was that moderate alcohol intake may be beneficial for postmenopausal women and that beer and wine have a higher protective effect on BMD compared to spirits, suggesting that beverage constituents other than alcohol may contribute to bone health. The relationship between light to moderate alcohol consumption with higher BMD is supported by observational cross-sectional studies (3, 44, 45), although other researchers have failed to find a significant association (10). However, there is a lack of scientific evidence from long-term intervention studies on beer consumption for comparison with the results of the present study.

Moreover, as pointed out by Godos et al. (42), little evidence is available for the impact of variables such as age, the evaluated skeletal site, duration of exposure to alcohol, and the pattern of drinking (46). Discrepant results between studies on alcohol and bone health could be related to differences in factors such as age and gender. The participants in the present study were in relatively early postmenopause, when bone loss and accelerated bone turnover arising from estrogen deficiency tends to be high (4). The age factor could explain why our results differed from those of the Framingham Offspring Cohort Study, which included older women with a mean age of 62.5 as well as men, who are expected to have lower rates of bone turnover and consequently, bone loss (11).

On the other hand, beer has been described as a rich source of dietary silicon (47). Ingestion of silicon-containing foods stimulates human osteoblasts and osteoblast-like cells to secrete type I collagen, which is involved in bone cell maturation and bone formation and enhances the calcification of the bone matrix. The incorporation of silicon in calcium phosphate bioceramics was also found to improve bone formation (15). In a randomized, placebo-controlled 12-month trial with osteopenic postmenopausal women, supplementation with 6 and 12 mg of choline-stabilized orthosilicic acid (ch-OSA) together with calcium/vitamin D3 resulted in higher PINP levels than the placebo, and a maintenance of lumbar and femoral BMD (48). In the current study, both beer interventions, with and without ethanol, increased the levels of bone formation markers, particularly PINP, which could be explained by the ingestion of silicon, an intrinsic component of beer. The apparent non-effect on bone mass could be attributed to the particularly rapid bone turnover in the early postmenopausal period, when the acceleration of bone resorption renders antiresorptive therapies especially useful. Conversely, in older women or in males, who experience a slower rate of bone loss and bone turnover, a therapeutic agent with moderate effects on bone formation would probably be more effective. We did not observe a decrease in bone resorption related to beer consumption and the slight increase in bone forming markers would be insufficient to prevent the negative imbalance in bone remodeling. In contrast, in previous studies including males and older postmenopausal women, moderate alcohol consumption was found to exert a positive effect on bone mass (6). Clearly, when the effect of moderate beer intake is analyzed, both the age and gender of the consumer need to be considered.

The phytoestrogen content of beer arises from the use of hops (*Humulus lupulus* L.) in its elaboration. Beer is particularly rich in the weakly estrogenic IX, which after ingestion is biotransformed into 8-PN, one of the strongest phytoestrogens known (49–51). In the postmenopausal state, circulation levels of estradiol fall considerably, and estrogen receptors in bones are downregulated. Dietary plant-derived phytoestrogens can induce the expression of these receptors and target specific estrogen receptor actions (52). Although more well-designed randomized clinical trials are still required, three recent meta-analyses restricted to randomized controlled trials concluded that isoflavones can have a positive effect on bone health (53–55). In their review of 63 controlled trials, Sansai et al. (55) found an improvement in BMD in the lumbar spine, femoral neck, and distal radius in postmenopausal women associated with the intake of 54 mg/day of genistein and 600 mg/day of ipriflavone (synthetic isoflavone) (55). In contrast with these findings, and in accordance with the results of Levis et al. (56), who carried out a 2-year, randomized, double-blind clinical trial in which women in early postmenopause consumed 200 mg of soy isoflavones/day (56), we did not observe this beneficial effect of moderate beer consumption in our small cohort during the 2-year intervention. Again, this would suggest that a more potent antiresorptive effect is necessary to prevent bone loss in the early postmenopausal period. The mechanisms of action of the phytoestrogen content of beer and its impact on sex hormones remain unknown.

The impact of silicon on bone health is rendered more complex by the inhibition of its absorption and distribution by sex hormone levels (12). It has been suggested that hormonal factors may overwhelm any favorable effects of dietary silicon on bones in postmenopausal women (13). Conversely, a review published in 2013 found evidence that moderate silicon supplementation enhances bone mineralization and density, independently of other factors (15). Moreover, a single dose intervention study reported that estradiol status had no obvious influence on silicon absorption (57), although the results may have been influenced by the large variation in serum estradiol concentrations among pre-menopause women and young men. More research is needed to determine the synergistic relationship between estrogen and silicon and to better understand the role of silicon in the management of early postmenopausal osteoporosis. Beer constitutes an interesting food matrix in this line of research, as it is rich not only in silicon but also in phenolic compounds with a phytoestrogenic effect.

A wide range of polyphenols are found in beer (23). Known for their antioxidant and anti-inflammatory activity (58), polyphenols can also inhibit osteoclast formation induced by receptor activator of nuclear factor-κB ligand (59, 60). The reported protective effect of wine consumption on bones has been related to its phenolic content, although there is a lack of *in vivo* evidence for the underlying mechanism (61). In a randomized, placebo-controlled trial, postmenopausal women administered capsules containing the wine polyphenol resveratrol (75 mg, twice daily) experienced a slower rate of bone loss in the lumbar spine and femur, and a slight reduction in bone resorption (62).

To our knowledge, the present clinical trial is the first to study the impact of daily moderate beer consumption (with and without ethanol) on bone health in a postmenopausal population. Although some positive effects on bone formation markers were found after the two beer interventions, the results should be interpretated with caution. The main weakness of the study is the small sample size, which may lack the statistical power to identify all the effects. Other limitations are the non-randomized design, possible intra-variability of exposure due to phenolic metabolism by gut microbiota and differences in AB and NAB prenylflavonoid profiles, and self-selection bias, as participation was voluntary, based on recruitment through advertisements. Silicon and iso-α-acids content of AB and NAB was not quantified, but the same commercial brand was used, making composition profiles more comparable. Additionally, neither serum silicon nor total silicon intake from the diet was monitored.

In 2001, the NIH Consensus concluded that there is an urgent need for randomized controlled trials of combination therapy, which includes pharmacological, dietary, and lifestyle interventions (including muscle strengthening, balance training, management of multiple drug use, smoking cessation, psychological counseling, and dietary interventions) (1). The present study contributes new insights into the possible benefits of beer consumption for bone health in postmenopausal women and reveals the need for more research in this field.

## Conclusion

The effect of beer intake on bone strength depends on the age, sex, and hormonal status of the consumer, as well as the drinking pattern. In this pilot study, daily moderate AB and NAB consumption in early postmenopausal women seemed to increase bone formation markers but had no effect on bone resorption markers, suggesting a positive modulating effect on bone health in this cohort. In contrast, the intervention did not produce changes in BMD and TBS determined at 2-years of treatment. Long-term randomized clinical trials are needed with greater number of participants to evaluate the benefits of moderate beer consumption in an older population of osteopenic post-menopausal women, particularly those aged over 60 years, as well as in males. The effect of both alcoholic and non-alcoholic fractions should also be analyzed.

## Data availability statement

The raw data supporting the conclusions of this article will be made available by the authors, without undue reservation.

## Ethics statement

The studies involving human participants were reviewed and approved by Bioethics Commission of the University of Barcelona (Institutional Review Board: IRB 00003099). The patients/participants provided their written informed consent to participate in this study.

## Author contributions

AT-R, PP, RE, and RL-R: conceptualization. MT-S: methodology, formal analysis, investigation, data curation, and writing—original draft preparation. AT-R, JM, PP, RE, and RL-R: validation and writing—review and editing. RE and RL-R: supervision. RL-R: project administration and funding acquisition. All authors had read and agreed to the published version of the manuscript.
