# Supplementary material for: Effect of moderate beer consumption (with and without ethanol) on osteoporosis in early postmenopausal women: Results of a pilot parallel clinical trial
Source: Front Nutr. 2022 Nov 15;9:1014140. doi: 10.3389/fnut.2022.1014140 (PMC9705783; doi:10.3389/fnut.2022.1014140)
Supplement: Supplementary file 1 [file Table_1.DOCX]

Supplementary Material

**Supplementary Table 1.** Intragroup and intergroup analysis of anthropometric and clinical measurements during the intervention study

|  | **Control**  **(n = 10)** | **AB**  **(n = 15)** | **NAB**  **(n = 6)** | ***p*-value^1^** |
| --- | --- | --- | --- | --- |
| BMI, kg/m^2^ |  |  |  |  |
| Baseline | 26.5 (25.3-32.5) | 26.5 (23.1-28.6) | 25.3 (24.7-29.0) | 0.595 |
| 12 months | 28.4 (25.5-33.2) | 28.1 (21.6-28.8) | 24.9 (24.8-28.2) | 0.291 |
| 24 months | 27.5 (25.1-32.0) | 27.3 (21.4-28.8) | 25.2 (24.4-28.8) | 0.472 |
| p-value^2^ | 0.557 | 0.121 | 0.688 |  |
| WC, cm |  |  |  |  |
| Baseline | 90.0 (85.5-100.0) | 88.7 (79.5-96.4) | 84.5 80.3-90.1) | 0.588 |
| 12 months | 91.1 (84.0-103.0) | 89.0 (82.0-96.4) | 82.3 (78.0-83.2) | 0.208 |
| 24 months | 94.0 (82.0-98.5) | 94.0 (83.0-99.0) | 85.3 (77.0-92.0) | 0.641 |
| p-value^2^ | 0.723 | 0.302 | 0.625 |  |
| Body fat mass, % |  |  |  |  |
| Baseline | 44.1 (40.2-45.1) | 42.7 (39.2-47.5) | 40.3 (39.1-48.2) | 0.900 |
| 12 months | 43.3 (39.3-44.6) | 44.0 (39.2-47.9) | 39.6 (36.4-41.5) | 0.409 |
| 24 months | 43.1 (38.0-46.1) | 43.1 (38.0-47.0) | 40.7 (37.6-42.0) | 0.671 |
| p-value^2^ | 0.922 | 0.417 | 1.000 |  |
| Fat mass index, kg/m^2^ |  |  |  |  |
| Baseline | 11.5 (9.6-15.3) | 11.2 (8.8-13.0) | 10.5 (9.3-12.3) | 0.636 |
| 12 months | 11.8 (9.6-13.8) | 12.1 (8.2-13.1) | 9.6 (8.5-11.0) | 0.354 |
| 24 months | 12.2 (9.7-13.4) | 11.6 (8.1-13.4) | 9.7 (8.7-11.7) | 0.592 |
| p-value^2^ | 0.695 | **0.030** | 0.688 |  |
| Lean mass index, kg/m^2^ |  |  |  |  |
| Baseline | 15.0 (14.5-17.2) | 14.2 (13.1-14.6) | 14.6 (14.0-16.5) | **0.034** |
| 12 months | 15.2 (14.6-17.1) | 14.2 (13.5-14.4) | 15.6 (14.4-16.4) | **0.008** |
| 24 months | 15.8 (14.8-17.7) | 14.9 (13.3-15.1) | 15.2 (14.5-16.3) | **0.043** |
| p-value^2^ | 0.432 | **0.013** | 1.000 |  |
| Physical activity, METS-min/day |  |  |  |  |
| Baseline | 840 (480-1146) | 552 (304-807) | 460 (396-601) | 0.238 |
| 12 months | 635 (517-1105) | 477 (266-731) | 748 (313-1124) | 0.273 |
| 24 months | 673 (535-1326) | 471 (207-709) | 764 (385-997) | 0.284 |
| p-value^2^ | 0.922 | 0.525 | 0.156 |  |
| Creatinine, mg/dL |  |  |  |  |
| Baseline | 0.71 (0.56-0.83) | 0.64 (0.59-0.75) | 0.68 (0.66-0.69) | 0.456 |
| 12 months | 0.73 (0.60-0.78) | 0.64 (0.61-0.74) | 0.72 (0.69-0.75) | 0.407 |
| 24 months | 0.79 (0.66-0.85) | 0.73 (0.64-0.87) | 0.86 (0.79-0.88) | 0.223 |
| p-value2 | 0.014 | 0.001 | 0.094 |  |
| Calcium (serum), mg/dL |  |  |  |  |
| Baseline | 9.3 (9.0-9.5) | 9.3 (9.0-9.5) | 9.3 (9.3-9.5) | 0.969 |
| 12 months | 9.3 (9.1-9.4) | 9.3 (9.1-9.5) | 9.0 (8.8-9.3) | 0.314 |
| 24 months | 9.4 (9.0-9.7) | 9.2 (8.9-9.4) | 9.2 (8.9-9.4) | 0.862 |
| p-value^2^ | 0.984 | 0.751 | 1.000 |  |
| PTH, ng/mL |  |  |  |  |
| Baseline | 63.0 (44.0-80.0) | 52.0 (46.0-69.0) | 66.5 (46.0-73.0) | 0.751 |
| 12 months | 65.0 (57.0-80.0) | 60.0 (52.0-68.0) | 70.0 (57.0-71.0) | 0.747 |
| 24 months | 66.0 (53.0-91.0) | 71.0 (64.0-94.0) | 66.5 (42.0-78.0) | 0.659 |
| p-value^2^ | 0.053 | **0.004** | 0.375 |  |
| 25-hydroxy-vitamin D, ng/mL |  |  |  |  |
| Baseline | 23.7 (20.6-26.5) | 25.4 (18.6-35.7) | 24.6 (14.1-38.6) | 0.743 |
| 12 months | 23.2 (21.6-24.4) | 24.8 (13.6-31.6) | 21.2 (13.6-26.5) | 0.710 |
| 24 months | 22.6 (20.2-23.7) | 25.2 (18.0-26.8) | 25.4 (22.4-28.2) | 0.620 |
| p-value^2^ | 0.846 | 0.241 | 0.675 |  |

AB: alcoholic beer; BMI: body mass index; ﻿NAB: non-alcoholic beer; PTH: parathyroid hormone; WC: waist circumference. Data are expressed as median values (Q1-Q3).

p-value^1^ refers to the intergroup comparisons by the Kruskal–Wallis test followed by Dunn’s test. p < 0.050 are statistically significant.

p-value^2^ refers to the difference between baseline and 24 months in each study arm. Matched-pair signed-rank test was used for statistical intragroup comparisons throughout the intervention. Sing-test of matched pairs was used for asymmetrically distributed variables.

**Supplementary Table 2.** Intragroup and intergroup analysis of dietary habits from food frequency questionnaire during the intervention study

|  | **Control**  **(n = 10)** | **AB**  **(n = 15)** | **NAB**  **(n = 6)** | ***p*-value^1^** |
| --- | --- | --- | --- | --- |
| Energy, kcal/day |  |  |  |  |
| Baseline | 2699 (2556-3022) | 2599 (2127-3138) | 2348 (2268,-682) | 0.320 |
| 12 months | 2682 (2384-2757) | 2478 (1946-3668) | 2583 (2297-3075) | 0.928 |
| 24 months | 2439 (2242-2636) | 2775 (1909-3712) | 2354 (2169-2781) | 0.611 |
| *p*-value^2^ | 0.160 | 0.600 | 1.000 |  |
| Carbohydrates, % kcal/day |  |  |  |  |
| Baseline | 31.4 (24.7-33.7) | 33.5 (29.2-38.9) | 37.4 (34.1-40.1) | 0.076 |
| 12 months | 33.6 (28.3-37.7) | 30.2 (26.6-36.9) | 37.9 (34.9-42.5) | 0.085 |
| 24 months | 34.1 (31.8-35.4) ^a^ | 28.9 (24.8-33.5) ^a^ | 40.9 (38.0-43.1) ^b^ | **<0.001** |
| *p*-value^2^ | 0.193 | **0.008** | 0.063 |  |
| Sugar, % kcal/day |  |  |  |  |
| Baseline | 17.1 (13.9-18.9) | 14.6 (11.3-16.9) | 19.7 (11.8-26.5) | 0.330 |
| 12 months | 18.0 (15.5-21.2) ab | 14.8 (10.7-18.0) b | 20.3 (16.9-24.2) a | 0.034 |
| 24 months | 16.5 (11.9-20.0) ab | 14.2 (10.9-16.9) b | 24.8 (16.1-25.9) a | 0.012 |
| *p*-value^2^ | 0.625 | 0.208 | 0.219 |  |
| Fiber, g/day |  |  |  |  |
| Baseline | 37.6 (33.2-44.3) | 36.6 (29.7-41.0) | 37.4 (33.7-37.8) | 0.829 |
| 12 months | 40.3 (33.1-44.5) | 33.5 (22.5-45.6) | 40.3 (39.2-41.1) | 0.589 |
| 24 months | 39.4 (34.9-44.5) | 29.6 (21.9-41.6) | 37.6 (34.1-38.6) | 0.246 |
| *p*-value^2^ | 0.492 | 0.169 | 0.679 |  |
| Protein, % kcal/day |  |  |  |  |
| Baseline | 20.4 (16.3, 20.9) | 19.2 (17.4, 21.8) | 18.1 (16.9, 20.4) | 0.781 |
| 12 months | 18.0 (16.5, 20.8) | 18.2 (15.0, 20.5) | 16.7 (16.4, 19.4) | 0.736 |
| 24 months | 17.3 (16.6, 20.6) | 17.7 (15.9, 20.0) | 17.2 (14.8, 18.4) | 0.691 |
| *p*-value^2^ | 0.492 | 0.302 | 0.313 |  |
| Total fat, % kcal/day |  |  |  |  |
| Baseline | 49.7 (44.6, 54.0) | 47.3 (37.4, 50.2) | 44.0 (41.3, 45.3) | 0.153 |
| 12 months | 46.0 (43.9, 54.1) | 47.2 (41.5, 51.9) | 41.9 (38.6, 49.4) | 0.424 |
| 24 months | 48.1 (46.5, 51.8) a | 51.5 (45.3, 53.8) a | 41.2 (37.6, 46.6) b | **0.037** |
| *p*-value^2^ | 0.695 | 0.073 | 0.313 |  |
| SFA, % kcal/day |  |  |  |  |
| Baseline | 13.8 (13.2, 15.0) | 12.7 (10.9, 14.5) | 12.5 (11.8, 14.1) | 0.176 |
| 12 months | 12.8 (11.4, 14.7) | 12.1 (11.3, 13.1) | 11.9 (10.1, 12.7) | 0.660 |
| 24 months | 12.6 (11.2, 13.3) | 12.8 (11.7, 13.9) | 10.6 (9.6, 11.6) | 0.134 |
| *p*-value^2^ | 0.006 | 0.169 | 0.156 |  |
| MUFAs, % kcal/day |  |  |  |  |
| Baseline | 21.8 (20.3, 27.6) | 23.3 (16.5, 26.8) | 20.3 (18.4, 24.0) | 0.469 |
| 12 months | 21.8 (20.4, 28.7) | 23.9 (19.2, 27.9) | 19.7 (16.8, 25.0) | 0.335 |
| 24 months | 24.3 (23.4, 26.1) | 24.5 (22.7, 28.9) | 19.8 (18.2, 21.4) | 0.079 |
| *p*-value^2^ | 0.770 | 0.107 | 0.844 |  |
| PUFAs, % kcal/day |  |  |  |  |
| Baseline | 7.6 (6.9, 8.2) | 6.6 (6.0, 8.4) | 6.7 (5.6, 8.8) | 0.440 |
| 12 months | 8.0 (7.3, 9.4) | 7.2 (5.9, 7.9) | 6.5 (6.0, 8.5) | 0.163 |
| 24 months | 7.8 (7.4, 8.9) | 7.3 (6.1, 8.2) | 6.8 (6.5, 7.7) | 0.091 |
| *p*-value^2^ | 0.557 | 0.359 | 1.000 |  |
| Alcohol, g/day |  |  |  |  |
| Baseline | 1.0 (0.0, 1.7) a | 7.7 (2.9, 9.2) b | 1.9 (0.6, 3.1) a | **<0.001** |
| 12 months | 0.6 (0.0, 0.8) a | 12.4 (11.6, 13.7) b | 3.9 (3.3, 4.6) a | **<0.001** |
| 24 months | 0.0 (0.0, 1.3) a | 12.4 (11.2, 12.7) b | 3.9 (3.3, 4.6) a | **<0.001** |
| *p*-value^2^ | 0.297 | **<0.001** | 0.313 |  |
| Calcium, mg/day |  |  |  |  |
| Baseline | 1365 (1090, 1679) | 1199 (935, 1552) | 1083 (824, 1334) | 0.405 |
| 12 months | 1122 (810, 1543) | 1108 (810, 1543) | 1108 (645, 1244) | 0.904 |
| 24 months | 915 (837, 1188) | 1251 (827, 1766) | 872 (638, 895) | 0.206 |
| *p*-value^2^ | 0.010 | 0.934 | 0.219 |  |
| Vitamin D, µg/day |  |  |  |  |
| Baseline | 6.1 (4.0, 9.8) | 6.4 (4.9, 8.3) | 6.3 (5.7, 7.0) | 0.995 |
| 12 months | 6.4 (5.1, 7.8) | 5.8 (5.3, 7.4) | 9.7 (5.8, 11.9) | 0.253 |
| 24 months | 6.4 (4.0, 8.0) | 8.4 (5.3, 10.8) | 5.9 (3.6, 7.1) | 0.143 |
| *p*-value^2^ | 1.000 | 0.107 | 0.438 |  |
| Total polyphenols, mg/day |  |  |  |  |
| Baseline | 1064 (770, 1419) | 753 (487, 853) | 830 (677, 1450) | 0.127 |
| 12 months | 1243 (810, 1562) | 844 (681, 973) | 1175 (1100, 1295) | 0.224 |
| 24 months | 1006 (589, 1455) | 742 (487, 958) | 1126 (1107, 1638) | 0.173 |
| *p*-value^2^ | 0.846 | 0.107 | 0.210 |  |

AB: alcoholic beer; ﻿NAB: non-alcoholic beer; SFA: saturated fatty acids; MUFAs: monounsaturated fatty acids; PUFAs: polyunsaturated fatty acids. Data are expressed as median values (Q1-Q3).

*p*-value^1^ refers to the intergroup comparisons by the Kruskal–Wallis test followed by Dunn’s test. p < 0.050 are statistically significant.

*p*-value^2^ refers to the difference between baseline and 24 months in each study arm. Matched-pair signed-rank test was used for statistical intragroup comparisons throughout the intervention. Sing-test of matched pairs was used for asymmetrically distributed variables.
